# Living Larvae in the Ear, and Methods for Their Destruction

**Published:** 1876-01

**Authors:** Charles H. Burnett

**Affiliations:** Surgeon in charge of the Philadelphia Infirmary for Diseases of the Ear


Living Larvæ in the Ear, and Methods for their Destruction. *By Charles H. Burnett, M.D., Surgeon in charge of the Philadelphia Infirmary for Diseases of the Ear.*—In some instances, usually in cases of chronic aural discharge, maggots develop in the auditory canal, and even in the tympanic cavity after the ear has been invaded by the fly.

Kuntzmann, Heine, and Blake have published accounts of the growth of maggots in the ear, and the latter authority has described minutely the apparatus with which these creatures maintain a hold in and lacerate the auditory canal and membrana tympani.

Having placed the living larvæ in a glass vessel containing a piece of raw meat and some warm water, Dr. Blake observed the motions and actions of the larvæ under the microscope.

He found that the apparatus by which the maggot makes and retains its hold is composed of a delicate horny frame-work, armed with two stout and horny hooks. By repeated extension and retraction of these hooks, brought about by a kind of telescopic motion of the bony framework, the animal pierces and tears the softest and warmest tissues it can lay hold upon, and hence it usually seeks the fundus of the auditory canal, and is sometimes found even in the tympanic cavity.

Larvæ of the Muscida sarcophaga (Blake and Gruber) and of the Muscida lucilia (Blake) have been found in the ear and watched through all stages of transformation. All accounts of finding these larvæ in the ear have shown that the organ attacked has been previously diseased, usually with a chronic purulent discharge, the odors of which attract the parent fly.

An apparent exception to this rule came under my notice not long since, in the Philadelphia Infirmary for Diseases of the Ear.

A young and intelligent woman stated that a day or two before her visit to the infirmary, while sitting sewing, she had been suddenly attacked with severe pain in a previously healthy ear.

She was obliged to discontinue her work on account of the severity of the pain, and to apply a poultice to the ear.

The next morning she felt a tickling and crawling sensation in the ear, and upon removing the poultice, three maggots crawled from the affected ear. From that time all pain and discomfort ceased. All these statements were fully corroborated by the patient's mother, who accompanied her.

Unfortunately for the perfect substantiation of all these statements of the patient, the maggots had been thrown away, and I can only give the statement as it was given to me; but I am disposed to believe that maggots were to be found in this case, and that they came out of the ear on account of the softer pabulum offered by the poultice.

This, however, is only conjecture, and whether maggots ever voluntarily leave the ear after having obtained lodgment there, can only be decided by future observation of their habits.

As I had considerable desire to find out a quick method of killing larvæ in the ear without injuring the organ attacked, as well as to observe their action, I made the following experiments:

A large fly, found upon the window-pane, was caught, and, by pressing, was forced to extrude about fifty live maggots, two millimetres long, and of a yellowish-white color. These were placed in a glass vessel, tightly corked, along with the dead parent fly, and nothing more. After twenty-four hours they were found to be still alive, thus showing their great tenacity of life. Some of them had crawled back again into the fly, and others crawled into her as I watched them.

I then placed a little piece of cold roast-beef, softened in water, into the glass vessel, for the maggots to live upon, and allowed the vessel to stand upon a table, at the ordinary temperature of the room.

Twenty-four hours later I found the maggots very active, and grown to be five millimetres long, with their alimentary canals stained by the brown juices of the roast meat.

In order to try tho effects of some easily-obtained fluids, innocuous to the ear, upon the maggots, I placed maggot No. 1 in a few drops of refined kerosene oil (Pratt's astral). It crawled repeatedly from the oil, and continued to live, though it was constantly thrust back again into the test-tube containing the fluid, and kept submerged in the kerosene. After a vain endeavor for a half-hour thus to kill the maggot, it was killed in another way.

Maggot No. 2 was placed in a saturated solution of salicylic acid (bleached, prepared by Hance Bros. & White, of this city); it died in half an hour.

Maggot No. 3 I placed in alcohol, where it died in five minutes.

Maggot No. 4 was placed in æther fortior (Squibb's), and was killed thereby in two minutes.

Maggots Nos. 5, 6, and 7 were placed in *a few drops of chloroform, which instantly killed them.* A few drops added to the squirming mass in the glass vessel killed them all instantly.

Chloroform-vapor forced through the Eustachian tube has been found fatal to the life of those creatures when they have penetrated to the middle ear.

An eighth specimen was placed in hydrant-water, which seems, as Dr. Roosa has also observed, to make them more lively at first, and they continued to live in it and work their savage hooks for an indefinitely long time.

Water appears not to have the slightest power to arrest their work when they have once gained a hold in the soft tissues of the ear. This may be due to the stimulating effects of the oxygen in the water.

I examined these maggots, and found that they were armed with an apparatus for holding on and tearing similar to that described by Blake.

The number of maggots found in the ear varies from a few, six in Kuntzmann's case, to the large number a single fly may deposit.

From the experiments just narrated, it seems probable that a few drops of chloroform instilled into the external auditory canal would kill all maggots found there, as well as those which have burrowed into the middle ear, without resorting to the use of chloroform-vapor through the Eustachian tube. If chloroform should be objected to in a case of larvæ in the ear, an almost equally potent means of killing the invaders may be found in ether, and, next to it, alcohol.

After the larvæ are killed it is often necessary to remove them from the auditory canal by means of forceps, so firm is the hold they gain upon the tissues they attack.

I observed, however, in those I examined a great tendency to leave the meat and migrate about the glass vessel, invariably in the end towards the mouth of the long glass test-tube in which they were confined, until at last the under surface of the cork was covered with them. This would naturally lead to the inference that in some cases where they attack the ear they may, for reasons unknown to us, let go their hold and escape from their confined position, perhaps in order to find earth in which to bury themselves.

In Kuntzmann's case the larvæ, when placed in a glass jar containing both earth and meat, preferred burying themselves in the earth to eating the meat. By some such desire on their part may be explained the fact that, in the case of the girl stated above, the maggots crawled from the ear to seek the poultice.

Why it is that in some cases so few maggots are found in the ear in comparison to the large number usually deposited by a fly elsewhere, when unmolested, may be due to the fact that the fly is interrupted when depositing her brood in or about the ear, or, as their extrusion is usually performed suddenly, some of the larvæ may fall short of the ear and perish.—*Med. Times.*

